# Genistein Induces Increase in Fluid pH, Na^+^ and HCO_3_^−^ Concentration, SLC26A6 and SLC4A4 (NBCe1)-B Expression in the Uteri of Ovariectomized Rats

**DOI:** 10.3390/ijms15010958

**Published:** 2014-01-10

**Authors:** Asma Chinigarzadeh, Nor Fadila Kasim, Sekaran Muniandy, Normadiah M. Kassim, Naguib Salleh

**Affiliations:** 1 Department of Physiology, Faculty of Medicine, University of Malaya, Lembah Pantai, Kuala Lumpur 50603, Malaysia; E-Mails: mehr_kimia2000@yahoo.com (A.C.); najeeb_98@hotmail.com (N.F.K.); 2 Department of Molecular Medicine, Faculty of Medicine, University of Malaya, Lembah Pantai, Kuala Lumpur 50603, Malaysia; E-Mail: sekaran@um.edu.my; 3 Department of Anatomy, Faculty of Medicine, University of Malaya, Kuala Lumpur 50603, Malaysia; E-Mail: normadiah_mk@um.edu.my

**Keywords:** genistein, SLC26A6, SLC4A4, uterine fluid

## Abstract

Genistein has been reported to stimulate luminal HCO_3_^−^ secretion. We hypothesized that genistein mediates this effect via SLC26A6 and SLC4A4 (NBCe1) transporters. Our study aimed to: investigate changes in uterine fluid pH, Na^+^ and HCO_3_^−^ concentration and expression of uterine SLC26A6 and NBCe1 under genistein effect. Ovariectomized adult female rats received 25, 50 and 100 mg/kg/day genistein for a week with and without ICI 182780. A day after the last injection, *in vivo* uterine perfusion was performed to collect uterine fluid for Na^+^, HCO_3_^−^ and pH determination. The animals were then sacrificed and uteri were removed for mRNA and protein expression analyses. SLC26A6 and NBCe1-A and NBCe1-B distribution were visualized by immunohistochemistry (IHC). Genistein at 50 and 100 mg/kg/day stimulates uterine fluid pH, Na^+^ and HCO_3_^−^ concentration increase. Genistein at 100 mg/kg/day up-regulates the expression of SLC26A6 and SLC4A4 mRNA, which were reduced following concomitant ICI 182780 administration. In parallel, SLC26A6 and NBCe1-B protein expression were also increased following high dose genistein treatment and were localized mainly at the apical membrane of the luminal epithelia. SLC26A6 and NBCe1-B up-regulation by genistein could be responsible for the observed increase in the uterine fluid pH, Na^+^ and HCO_3_^−^ concentration under this condition.

## 1. Introduction

Genistein has been reported to affect epithelial HCO_3_^−^ transport, and thus could influence the fluid pH. In the small intestine, genistein stimulates Cl^−^ and to a smaller extent, HCO_3_^−^ secretion [1]. In the airways [2], duodenum [3], jejunum [4], epididymis [5] and oesophagus [6], genistein was also found to stimulate HCO_3_^−^ secretion, which could affect pH of the luminal fluid. Epithelial HCO_3_^−^ secretion, which is regulated by hormones and paracrine factors, is mediated via the HCO_3_^−^ transporters such as Cl^−^/HCO_3_^−^ exchanger (SLC26A6) and Na^+^- HCO_3_^−^ co-transporter (SLC4A4). In the uterus, increased luminal fluid pH under estrogen influence has been reported to be partly due to increased luminal fluid HCO_3_^−^ secretion via SLC26A6 [7] and SLC4A4 [8] transporters.

SLC26A6 is a member of a large, conserved family of anion exchanger (SLC26) [9]. Human SLC26A6 was mapped to chromosome 3 and encodes a 738-amino acid (aa) protein [10]. Rodent SLC26A6 and human SLC26A6 share 78% of the aa identity [11]. Functional studies in *in vitro* expression systems have demonstrated that SLC26A6 mediates multiple anion exchange modes, including Cl^−^/HCO_3_^−^, Cl^−^/oxalate, Cl^−^/OH^−^ and Cl^−^/formate exchanges [12]. In the kidney proximal tubule, SLC26A6 is the major contributor of NaCl absorption [13], while in the doudenum, it participates in HCO_3_^−^ secretion in exchange with Cl^−^ absorption [14]. SLC26A6 plays an active role in epithelial HCO_3_^−^ secretion in the proximal portion of the pancreatic duct where HCO_3_^−^ is transported against the concentration gradient into the lumen [15].

Na^+^- HCO_3_^−^ co-transporter (NBC) belongs to the solute carrier 4 family (SLC4) [16]. Currently, there are five known members of Na^+^-coupled HCO_3_^−^ co-transporters (NBCe1, NBCe2, NBCn1, NBCn2 and NDCBE). Two of these (NBCe1, NBCe2) are electrogenic while three others are electroneutral [17]. NBCe1 (SLC4A4) and NBCe2 (SLC4A5) transport net negative charge [18]. NBCe1 consists of NH_2_-terminal variants *i.e.*, NBCe1-A (kNBC) and NBCe1-B (pNBC) [19]. NBCe1-A mediates basolateral electrogenic sodium-base transport for example in the kidney proximal tubule and is critically required for transepithelial HCO_3_^−^ absorption [20]. Meanwhile, in the secretory epithelia such as pancreatic duct [21], gastrointestinal tract [22] and parotid-salivary ducts [23], NBCe1-B is abundantly expressed and is responsible for basolateral HCO_3_^−^ uptake for secretion at the apical membrane. In the corneal endothelia, NBCe-1, which was expressed at both the apical [24] and basolateral membranes [25], is responsible for HCO_3_^−^ secretion into the aqueous humor.

Given that genistein is a weak estrogen and is capable of binding to the estrogen receptor, which is abundantly expressed in the uterus [26], we hypothesized that genistein could produce similar effect to estrogen in causing an increase in the pH, Na^+^ and HCO_3_^−^ concentration of the uterine luminal fluid via up-regulating the expression of HCO_3_^−^ transporters including SLC26A6 and SLC4A4. The aim of this study is therefore to investigate changes in these uterine fluid parameters under genistein effect as well as to investigate changes in the expression and distribution of SLC26A6 and SLC4A4 (NBCe1-A and NBCe1-B) in the uterus in ovariectomized rats receiving genistein.

## 2. Results and Discussion

### 2.1. Results

#### 2.1.1. Uterine Fluid pH, Na^+^ and HCO_3_^−^ Concentration Following Genistein Treatment

**Figure 1.**
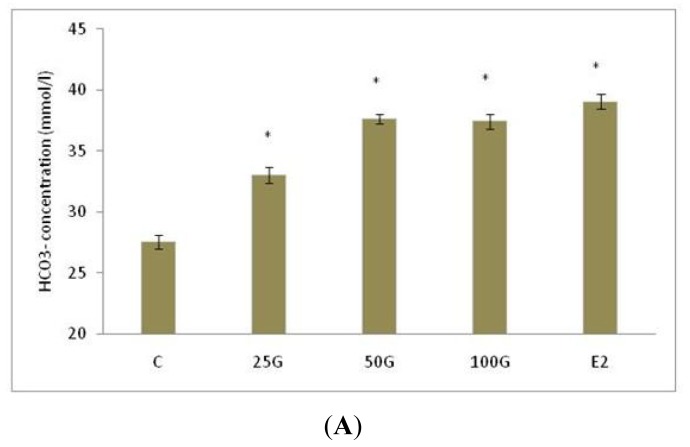

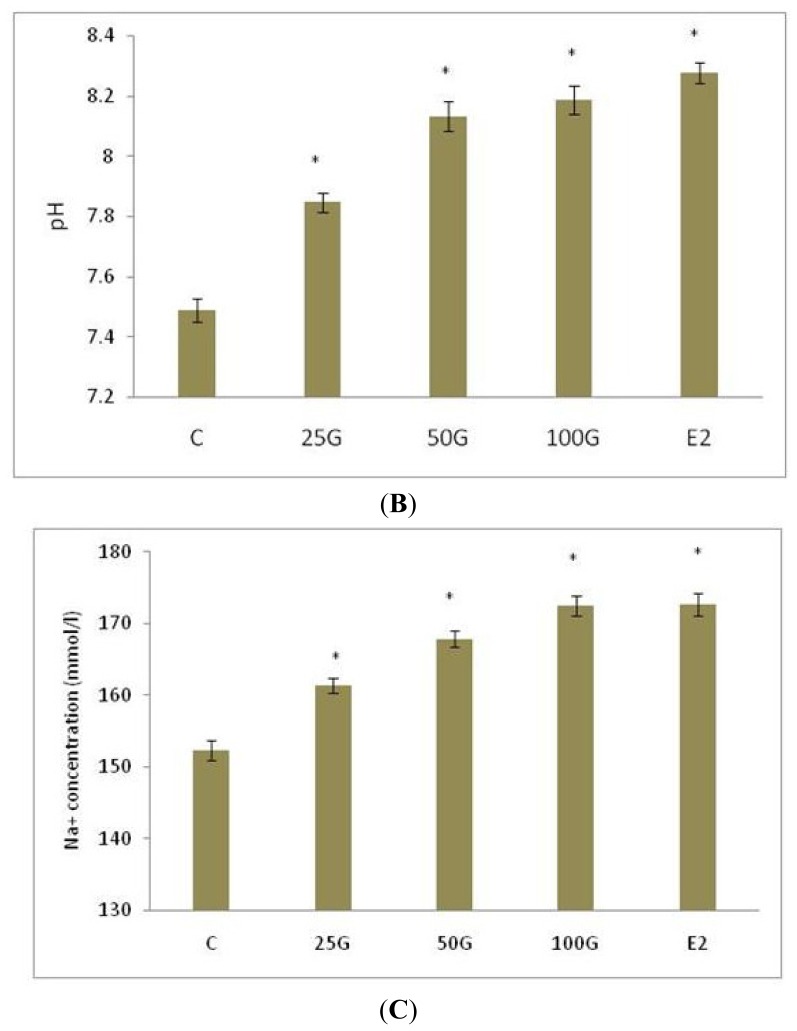
The effect of genistein at 25, 50 and 100 mg/kg/day on the (**A**) HCO_3_^−^; (**B**) pH and (**C**) Na^+^ concentration of the uterine luminal fluid in the ovariectomized SD rats. An increase in pH, Na^+^ and HCO_3_^−^ concentration were observed with increasing doses of genistein. The pH and HCO_3_^−^ concentration were the highest following treatment with 50 and 100 mg/kg/day genistein while Na^+^ concentration was the highest following treatment with 100 mg/kg/day genistein. Changes in pH and HCO_3_^−^ concentration following treatment with 50 and 100 mg/kg/day genistein were not statistically significant as compared to estrogen treatment. C: control; 25G: 25 mg/kg/day genistein; 50G: 50 mg/kg/day genistein; 100G: 100 mg/kg/day genistein; E2; estrogen. *n* = 6 rats per treatment group; * *p* < 0.05 as compared to control.

Figure 1A shows the HCO_3_^−^ concentration changes, Figure 1B shows the pH changes and Figure 1C shows the Na^+^ concentration changes following treatment with various doses of genistein at 25, 50 and 100 mg/kg/day. Our findings indicate that there was an increase in the pH, Na^+^ and HCO_3_^−^ concentration with increasing doses of genistein. Treatment with 25, 50 and 100 mg/kg/day genistein caused alkaline uterine fluid. The pH achieved following treatment with 100 mg/kg/day genistein was 8.29 ± 0.7 which was approximately similar to the pH achieved following estrogen treatment (8.32 ± 0.95). Meanwhile, treatment with 25, 50 and 100 mg/kg/day genistein also caused an increase in Na^+^ and HCO_3_^−^ concentration as compared to control, while changes in these electrolytes concentration following treatment with 100 mg/kg/day genistein was not significant as compared to the estrogen-treated group.

#### 2.1.2. SLC26A6 and SLC4A4 mRNA Expression

**Figure 2.**
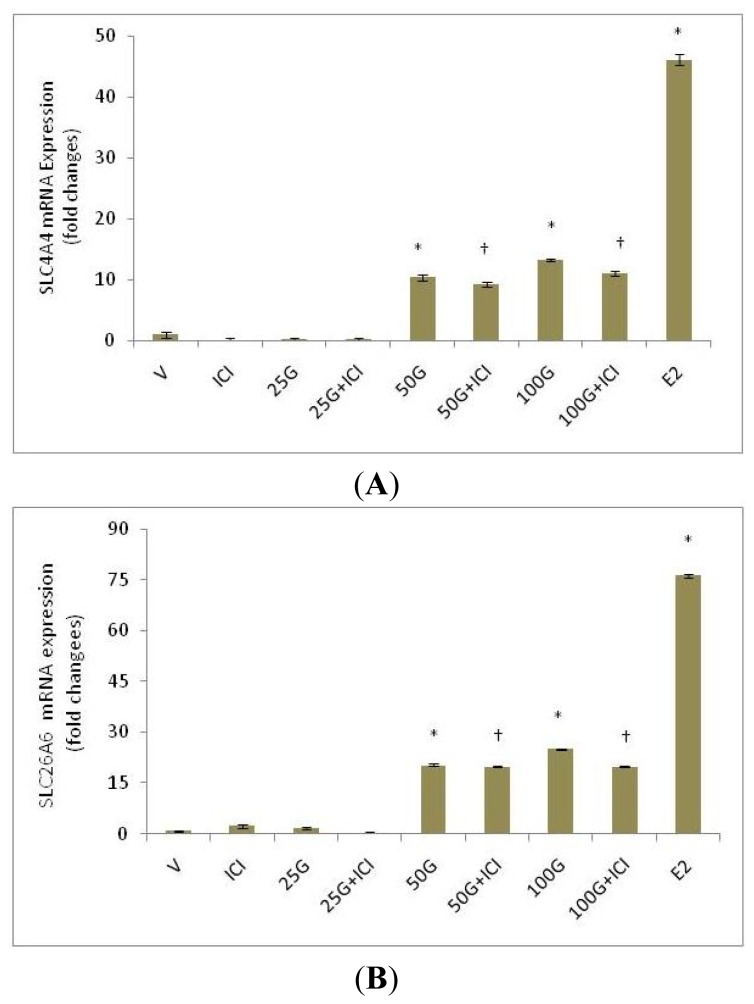
Fold changes in the mRNA expression of (**A**) SLC26A6 and (**B**) SLC4A4 in the whole uterine tissue homogenates from rats treated with various doses of genistein (25, 50 and 100 mg/kg/day). An increase in the mRNA expression was observed with increasing doses of genistein. The maximum expression of both transporters’ mRNA was observed following treatment with 100 mg/kg/day genistein and this was significantly lesser than estrogen treatment. Concomitant administration of ICI 182780 caused a significant decrease in the expression of both transporters’ mRNA. SLC26A6 mRNA expression exceed SLC4A4. V: vehicle (peanut oil); ICI: ICI 182780; 25G: 25 mg/kg/day genistein; 50G: 50 mg/kg/day genistein; 100G: 100 mg/kg/day genistein; E2; estrogen. *n* = 6 rats per group; * *p* < 0.05 as compared to control; ^†^ *p* < 0.05 as compared to without ICI.

Figure 2 shows fold changes in the expression of SLC26A6 and SLC4A4 mRNA in the uteri of ovariectomized rats treated with 25, 50 and 100 mg/kg/day genistein. Our findings indicate that genistein treatment caused an increase in the expression of both transporters’ mRNA. Following treatment with 100 mg/kg/day genistein, the expression of SLC26A6 exceeds SLC4A4 by more than two folds, and these were significantly lower than following estrogen treatment. ICI 182780 administration resulted in a significant decrease in the expression of these transporters’ mRNA, suggesting that estrogen receptor binding is necessary for genistein-induced up-regulation of SLC26A6 and SLC4A4 mRNA.

#### 2.1.3. SLC26A6 and SLC4A4 (NBCe1-A and NBCe1-B) Protein Expression

**Figure 3.**
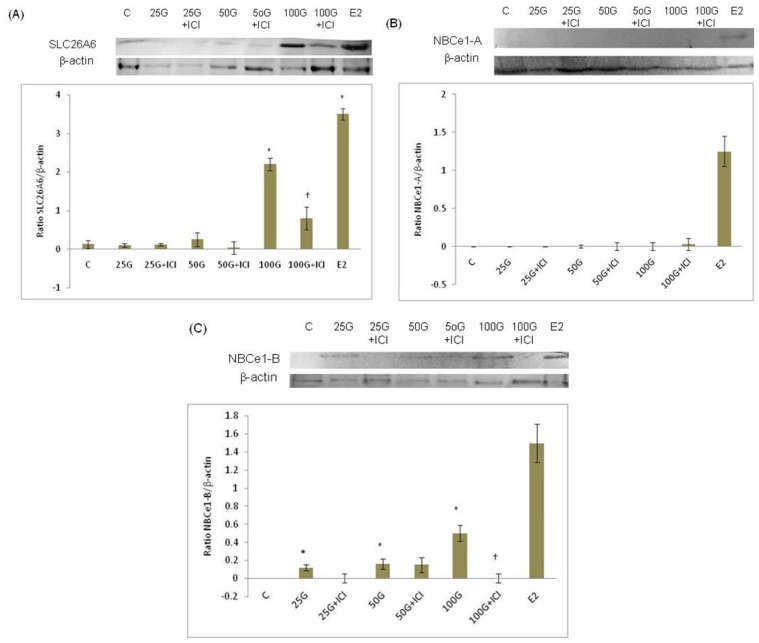
Protein expression analyses of (**A**) SLC26A6; (**B**) NBCe1-A; and (**C**) NBCe1-B of the whole uterine homogenates from rats treated with various doses of genistein (25, 50 and 100 mg/kg/day). An increase in SLC26A6 and NBCe1-B protein expression was observed with increasing doses of genistein, with the maximum expression occur following treatment with 100 mg/kg/day genistein, Concomitant administration of ICI 182780, an estrogen receptor blocker significantly reduced the expression of both transporters’ protein under genistein effect. The expression of SLC26A6 and NBCe1-B following high dose (100 mg/kg/day) genistein treatment were significantly lower than estrogen treatment (positive control). Higher expression of SLC26A6 was observed as compared to NBCe1-B following 100 mg/kg/day genistein treatment. NBCe1-A protein was however not expressed in the uterus under genistein effect and was minimally expressed following estrogen treatment. C: control; ICI: ICI 182780; 25G: 25 mg/kg/day genistein; 50G: 50 mg/kg/day genistein; 100G: 100 mg/kg/day genistein; E2; estrogen. *n* = 6 rats per group; * *p* < 0.05 as compared to control; ^†^ *p* < 0.05 as compared to without ICI.

In Figure 3A, the expression of SLC6A6 protein was increased following 100 mg/kg/day genistein treatment, although this was lower than estrogen treatment. Figure 3B shows that NBCe1-A protein was not expressed in the uterus following genistein treatment and was mildly expressed following estrogen treatment. Meanwhile, in Figure 3C, the expression of NBCe1-B was the highest following treatment with 100 mg/kg/day genistein, however was significantly lower than estrogen treatment. Administration of ICI 182780 resulted in reduced expression of SLC26A6 and NBCe1-B, which indicates that estrogen receptor binding is necessary for genistein-induced up-regulation of SLC26A6 and NBCe1-B proteins.

#### 2.1.4. SLC26A6, NBCe1-A and NBCe1-B Protein Distribution

**Figure 4.**
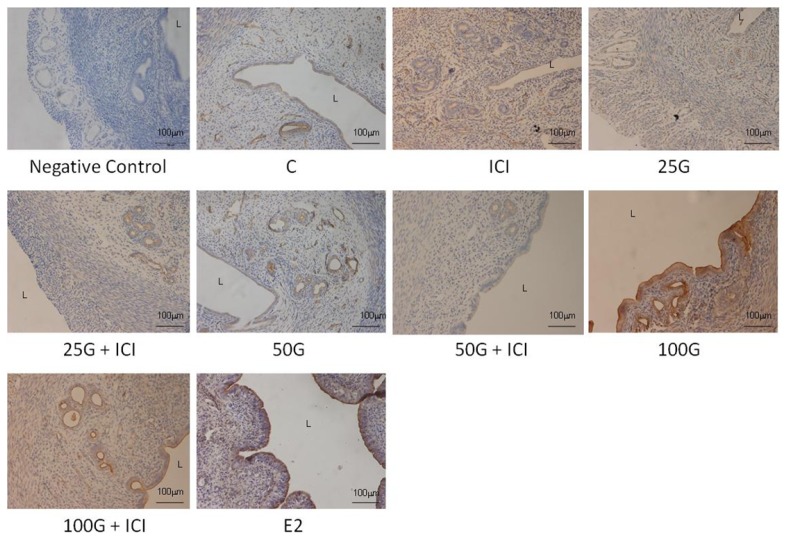
The distribution of SLC26A6 in the uterus of rats receiving various genistein doses (25, 50 and 100 mg/kg/day with and without the presence of ICI 182780, an estrogen receptor antagonist). An increase in SLC26A6 immunostaining intensity was seen at the apical membrane of the luminal and glandular epithelia with increasing doses of genistein. The intensity was reduced following concomitant administration of ICI 182780. High apical intensity was observed in the estrogen treated group (positive control). C: control; ICI: ICI 182780; 25G: 25 mg/kg/day genistein; 50G: 50 mg/kg/day genistein; 100G: 100 mg/kg/day genistein; E2: estrogen. *n* = 4 rats per group. The images were taken at 20× magnification. L: lumen.

**Figure 5.**
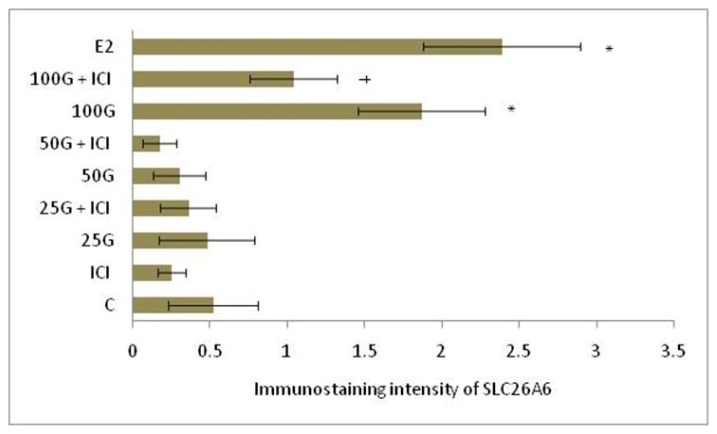
Quantitative evaluation of immunostaining intensity of SLC26A6 in the luminal epithelia following treatment with 25, 50 and 100 mg/kg/day genistein and estrogen by NIS-AR Element program. The intensity was the highest following treatment with estrogen, followed by 100 mg/kg/day genistein. C: control; ICI: ICI 182780; 25G: 25 mg/kg/day genistein; 50G: 50 mg/kg/day genistein; 100G: 100 mg/kg/day genistein; E2: estrogen. * *p* < 0.05 as compared to control; ^†^ *p* < 0.05 as compared to without ICI.

**Figure 6.**
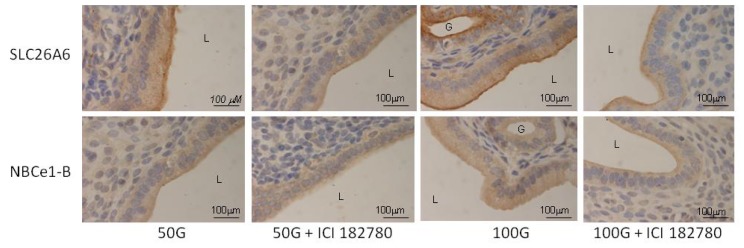
High magnification images (40×) shows the distribution of SLC26A6 and NBCe1-B at the apical and basolateral membranes of the luminal epithelia in rat receiving high doses genistein treatment (50 and 100 mg/kg/day with and without the presence of ICI 182780, an estrogen receptor antagonist). High SLC26A6 immunostaining intensity was seen following 100 mg/kg/day genistein treatment, which was reduced following concomitant administration of ICI 182780. Moderate NBCe1-B immunostaining intensity was observed at the apical membrane of the luminal epithelia following treatment with 100 mg/kg/day genistein. Meanwhile, the absence of immunostaining was seen at the basolateral membrane. ICI: ICI 182780; 50G: 50 mg/kg/day genistein; 100G: 100 mg/kg/daygenistein. L: lumen; G: gland.

**Figure 7.**
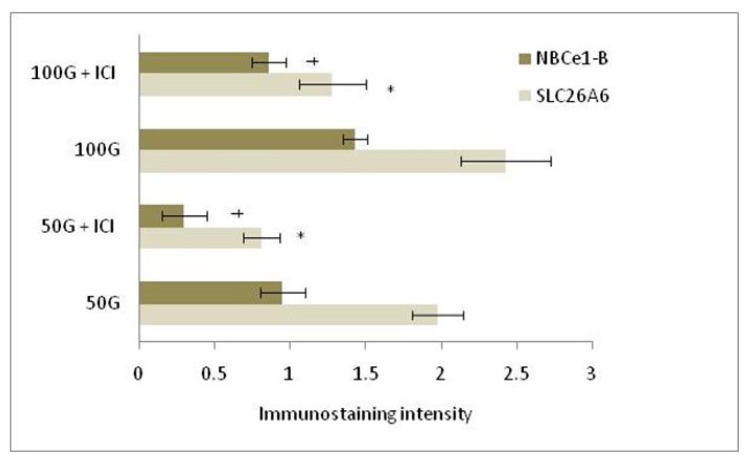
Quantitative analysis of immunostaining intensity of SLC26A6 and NBCe1-B at the apical membrane of the luminal epithelia following treatment with 50 and 100 mg/kg/day genistein by NIS-Element AR program. The intensity for both transporters was the highest at the apical membrane following 100 mg/kg/day genistein treatment. ICI: ICI 182780; 50G: 50 mg/kg/day genistein; 100G: 100 mg/kg/day genistein. * *p* < 0.05 as compared to control; ^†^ *p* < 0.05 as compared to without ICI.

In Figures 4 and 5, immunostaining intensity of SLC26A6 was the highest following treatment with 100 mg/kg/day genistein. Digital image analyses indicate that there was no significant difference in the intensity between this treatment group and estrogen treatment (positive control). Concomitant ICI 182780 administration resulted in a significant decrease in SLC26A6 immunostaining, the intensity indicating that the genistein effect is mediated via estrogen receptor binding. Moderate intensity was observed following treatment with 50 mg/kg/day genistein, which was also reduced in the presence of ICI 182780. The highest intensity of SLC26A6 immunostaining was seen at the apical membrane in the group receiving 100 mg/kg/day genistein, which was reduced in the presence of ICI 182780 (Figures 6 and 7). Meanwhile, no staining was observed at the basolateral membrane in these treatment groups (50 and 100 mg/kg/day genistein).

**Figure 8.**
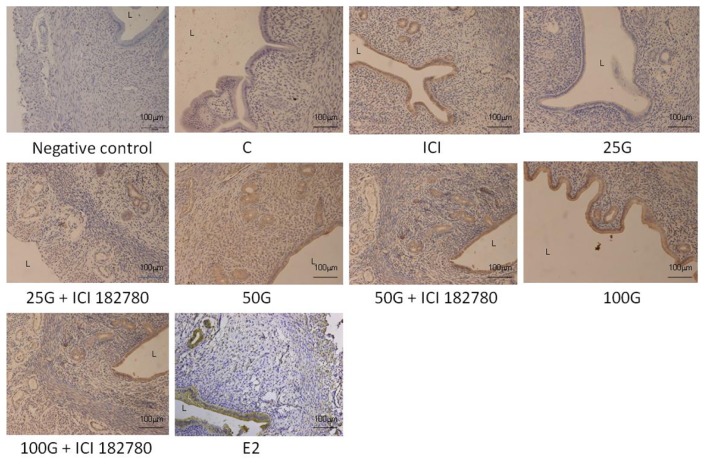
The distribution of NBCe1-B in the uterus of rats treated with various doses of genistein (25, 50 and 100 mg/kg/day with and without the presence of ICI 182780, an estrogen receptor antagonist). An increase in intensity of NBCe1-B immunostaining was seen mainly at the apical membrane of the luminal and glandular epithelia with increasing doses of genistein. The highest intensity was observed following treatment with estrogen followed by 100 mg/kg/day genistein. Concomitant administration of ICI 182780 caused a decrease in immunostaining intensity. C: control; ICI: ICI 182780; 25G: 25 mg/kg/day genistein; 50G: 50 mg/kg/day genistein; 100G: 100 mg/kg/day genistein; E2; estrogen. *n* = 4 rats per group. Images were taken at 20× magnification. L: lumen.

**Figure 9.**
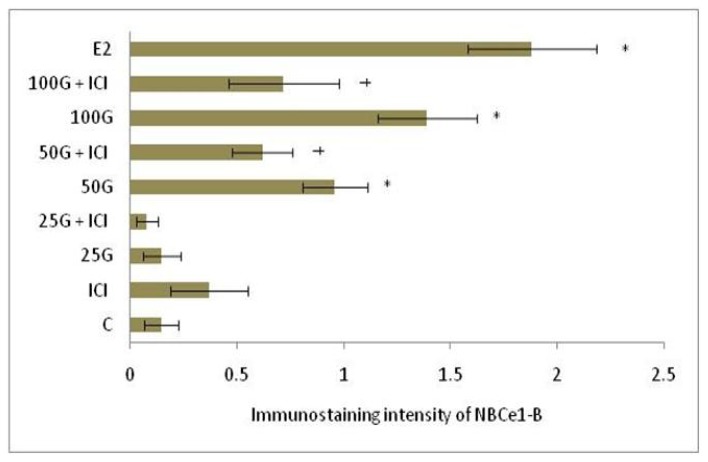
Quantitative analysis of immunostaining intensity of NBCe1-B in the endometrium following treatment with 25, 50 and 100 mg/kg/day genistein and estrogen (positive control) by NIS-Element AR program. The intensity was the highest following estrogen treatment followed by 100 mg/kg/day genistein. C: control; ICI: ICI 182780; 25G: 25 mg/kg/day genistein; 50G: 50 mg/kg/day genistein; 100G: 100 mg/kg/day genistein; E2: estrogen. * *p* < 0.05 as compared to control; ^†^ *p* < 0.05 as compared to without ICI.

**Figure 10.**
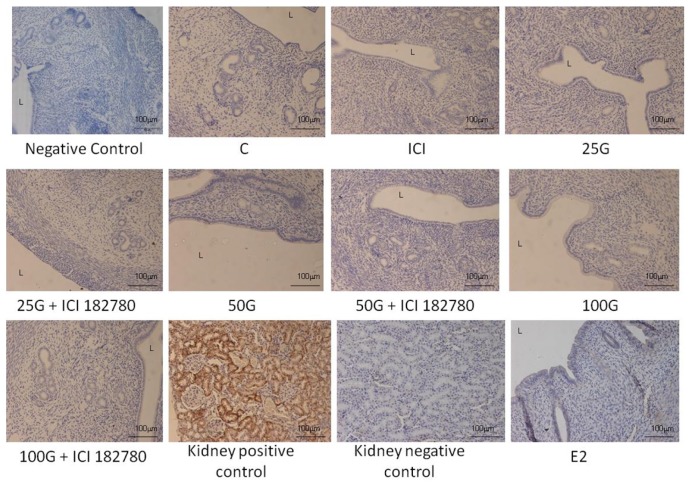
The distribution of NBCe1-A in the uterus of rats treated with various doses of genistein (25, 50 and 100 mg/kg/day with and without the presence of ICI 182780, an estrogen receptor antagonist). NBCe1-A was not expressed in the uterus following genistein treatment. The kidney was used as a positive control where intense immunostaining was seen at the basolateral membrane. Low staining intensity was seen at the apical membrane of the luminal epithelia following estrogen treatment. C: control; ICI: ICI 182780; 25G: 25 mg/kg/day genistein; 50G: 50 mg/kg/day genistein; 100G: 100 mg/kg/day genistein; E2: estrogen. *n* = 4 rats per group. The images were taken at 20× magnification. L: lumen.

In Figures 8 and 9, NBCe1-B immunostaining was the highest following treatment with 100 mg/kg/day genistein and the intensity was reduced in the presence of ICI 182780. In Figures 6 and 7, NBCe1-B expression could be seen at the apical membrane of the luminal epithelia following 100 mg/kg/day genistein treatment while there was lack of staining at the basolateral membrane. No staining for NBCe1-A was observed following low, moderate and high dose genistein treatment indicating that this NBCe1 variant was not expressed in the uterus under this condition (Figure 10).

### 2.2. Discussion

To the best of our knowledge, this study is the first to describe changes in the pH, Na^+^ and HCO_3_^−^ concentration of the uterine luminal fluid and the expression of SLC4A4 and SLC26A6 in the uteri of ovariectomized rats treated with genistein. Under high dose genistein effect, the increase in uterine fluid HCO_3_^−^ concentration results in alkaline uterine fluid with the pH almost similar to estrogen treatment. Our findings indicate that the expression of SLC26A6 was increased following treatment with high doses genistein (50 and 100 mg/kg/day), mainly at the apical membrane of the luminal epithelia. Additionally, the expression of NBCe1-B but not NBCe1-A was also increased at the apical membrane of the luminal epithelia following treatment with 100 mg/kg/day genistein. These findings support our functional data where the increase in luminal fluid Na^+^ and HCO_3_^−^ concentration could be mediated via the apically located SLC26A6 and NBCe1-B.

Following treatment with 100 mg/kg/day genistein, the concentration of uterine luminal fluid Na^+^ and HCO_3_^−^ was approximately 1.5 times higher than following 25 mg/kg/day genistein treatment. The concentration achieved were almost similar to that achieved following 0.8 × 10^−4^ mg/kg/day estrogen treatment, indicating that high dose genistein produced similar effect to estrogen in causing an increase in uterine fluid Na^+^ and HCO_3_^−^ content. Genistein effect was mediated via estrogen receptor (ER) as evidenced from ICI 182780 inhibition. ER is abundantly found in the uterus, consist of ER-α and ER-β with higher expression of the latter than the former [27]. Estrogen receptor mediated genistein effect on SLC26A6 and SLC4A4 expression was confirmed from down-regulation of these transporters’ mRNA and protein following concomitant ICI 182780 administration.

Our findings were supported by observations in several other tissues. Genistein has been reported to induce HCO_3_^−^ secretion in the duodenum [3], jejunum [4], epididymis [5] and airways [2]. While most studies implicated Cystic Fibrosis Transmembrane Regulator (CFTR) as a channel responsible for HCO_3_^−^ efflux under genistein influence [6,28], the involvement of Cl^−^/HCO_3_^−^ exchanger (SLC26A6) is equally important in mediating HCO_3_^−^ extrusion into the lumen [29]. SLC26A6 has been reported to mediate HCO_3_^−^ secretion in the duodenum [29] as well as in the proximal portion of the pancreatic duct, whereas in the distal portion [15], SLC26A6 together with CFTR are involved in mediating HCO_3_^−^ secretion against a high intraluminal concentration gradient [30]. In view of this, up-regulation of SLC26A6 expression by high dose genistein in the uterus could be responsible for the increase in uterine HCO_3_^−^ secretion with a subsequent increase in pH.

The electrogenic SLC4A4 (NBCe1) consists of two NH_2_-terminal variants *i.e.*, NBCe1-A and NBCe1-B, which expression can be detected by the antibodies raised against these specific amino termini. In view that common gene encodes NBCe1-A and NBCe1-B, therefore their mRNA sequences are similar. Identification of NBCe1-A and NBCe1-B variants could only be made from analyses of their proteins expression. Our findings indicate that the expression of SLC4A4 mRNA under high dose genistein (100 mg/kg/day) was approximately four (4) fold lower than following estrogen treatment. Under high dose genistein effect, the expression of SLC4A4 mRNA was relatively lower than SLC26A6 mRNA, suggesting that SLC4A4 plays a minor role in uterine fluid HCO_3_^−^ secretion under this condition.

Meanwhile, protein expression analyses and immunostaining revealed that only NBCe1-B but not NBCe1-A was expressed at the apical membrane of the luminal and glandular epithelia under high dose (100 mg/kg/day) genistein effect. These findings indicate that genistein-induced up-regulation of NBCe1-B might be involved in the increase in uterine fluid Na^+^ and HCO_3_^−^ secretion. The absence of NBCe1-A in the uterus was expected since this variant was found predominantly in the kidney where it participates in luminal HCO_3_^−^ reabsorption [20]. Meanwhile, NBCe1-B has been reported to participate in Na^+^ and HCO_3_^−^ efflux in the pancreatic ductal epithelia [21] and in the corneal endothelia [24], supporting the notion that this transporter could be involved in uterine luminal fluid Na^+^ and HCO_3_^−^ secretion.

Our findings have important translational inference to the female reproduction. High dose phytoestrogen consumption has been reported to reduce fertility in the sheep [31] and captive cheetahs [32] however the effect on human fertility is still unknown. Additionally, the mechanism underlying genistein-induced subfertility or infertility in these animal species remain elusive. A recent finding by Salleh *et al.*, [33] showed that genistein at 50 and 100 mg/kg/day can cause excessive accumulation of fluid in the uterine lumen in rats, which suggest that excessive fluid amount could be responsible for the adverse genistein effect on fertility. In this study, we have further shown that in addition to causing fluid secretion, genistein, in the absence of sex-steroids can stimulate Na^+^ and HCO_3_^−^ secretion as well increased the pH of uterine fluid in the same animal model. Interference of the fluid and electrolyes content may compromise successful implantation. Precise control of the uterine fluid pH and electrolytes are crucial for sperm transport, capacitation and acrosomal reaction, fertilization, embryo transport and implantation [34]. High dose genistein may interfere with these processes, thus may lead to infertility.

While our findings could have important implication to the female of the reproductive age, the ovariectomised model used in this study could also represent the post-menopausal condition. Changes in the fluid pH and electrolytes composition of the uterus following menopause are not well understood. There is currently lack of information with regards to the significance of uterine fluid during this period, which may function as lubricant to preserve the normal uterine environment and delaying uterine atrophy [35]. As genistein is widely consumed as a dietary supplement by the post-menopausal women, therefore this effect might be useful to prevent post-menopausal uterine atrophy.

## 3. Experimental Section

### 3.1. Animal Preparation and Hormone Treatment

Three month-old adult female Sprague-Dawley (SD) rats, weighing approximately 225 g were housed in a clean and well ventilated animal room with standardized housing conditions (lights on 12 h from 06:00 to 18:00 h: room temperature at 24 °C; with 5–6 animals per cage). The animals had free access to soy-free Harlan diet and water free from dissolving endocrine-disrupting chemicals (EDCs) since the time of weaning. All procedures were approved by the Faculty of Medicine, Animal Care and Use Committee, University of Malaya, Kuala Lumpur, Malaysia with the ethics number: 2013-07-15/FIS/R/NS. Genistein (G-6055) was purchased from LC Laboratories (Woburn, MA, USA) with more than 99% purity which appears as crystalline powder with light yellow color.

Bilateral ovariectomy was performed under isoflurane anesthesia at least ten days prior to drug treatment to eliminate the effect of endogenous sex-steroids [36]. After surgery, the animals were given intramuscular injection of 0.1 mL of Kombitrim antibiotic to prevent post surgical wound infection. The animals were divided into seven (7) groups (*n* = 6 per group). Group 1 was treated for seven (7) days with peanut oil (vehicle), Groups 2 to 7 received subcutaneous genistein at the following doses: 25, 50 and 100 mg/kg/day for seven (7) consecutive days with and without ER antagonist, ICI 182780 at 100 μg/kg/day. A positive control group was treated with 0.8 × 10^−4^ mg/kg/day estrogen also for 7 days. The drugs were dissolved in DMSO and were then administered via subcutaneous injection behind the neck cuff.

### 3.2. *In Vivo* Uterine Perfusion, pH and Electrolytes Concentration Analyses

*In vivo* uterine perfusion was performed according to method by Salleh *et al.*, [36]. In brief, a day after the last drug treatment, the animals were anesthetized with intraperitoneal (i.p.) injection of xylazine HCl (8 mg/kg) and ketamine (80 mg/kg). The animal was placed on a heat pad to maintain a constant body temperature at 37 °C. An incision was made at both flanks to expose the abdominal cavity and an in-going tube (fine polythene tubing ID 0.38 mm, OD 1.09 mm, pre-filled with perfusate) was inserted at the distal end of the uterine horns. A midline anterior incision was made in the anterior abdomen to insert an out-going tube which was tied *in situ* at the uterocervical junction. A syringe-driven infusion pump (Harvard Apparatus, Holliston, MA, USA) was used to deliver perfusion medium into the uterine lumen at a constant rate of 0.75 μL/min. The in-going tube, animal and out-going tube were placed at the same level to minimize the gravitational effect. The perfused fluid was collected into a small, pre-weighed polythene tubes with covered tops to minimize evaporation.

Perfusion was conducted over a period of 3 h. At the end of the experiment, the animals were sacrificed by cervical dislocation. The perfusate contains the following: 110.0 mmol/L NaCl, 14.3 mmol/L Na_2_HCO_3_, 1.0 mmol/L Na_2_HPO_4_, 15 mmol/L KCl, 0.8 mmol/L MgSO_4_, 10.0 mmol/L HEPES, 1.8 mmol/L CaCl_2_ and 5.5 mmol/L glucose at pH 7.34 which were selected to closely mimic the normal uterine fluid composition [37]. pH of the collected samples (usually less than 500 μL) was directly measured using HI 8424 NEW micropH meter from Hanna instrument (Smithfield, RI, USA). The collected samples were briefly exposed to air to equilibrate the dissolved CO_2_ with the atmosphere. HCO_3_^−^ concentration was determined by enzymatic assay using phosphoenolpyruvate carboxylase (PEPC; Vannas, Sweden) and malate dehydrogenase (MDH; Worthington Biochemical Corp, Lakewood, NJ, USA) in which the end product was measured by spectrophotometer at an absorbance wavelength of 405 or 415 nm, which was directly proportional to the HCO_3_^−^ concentration in the samples. Na^+^ concentration was measured directly using Ion Selective Electrode (ISE) (Fisher Scientific International Inc., Hampton, NH, USA), which was voltage-dependent on the levels of ion in the solution.

### 3.3. mRNA Quantification by Real Time PCR (qPCR)

Whole uterine tissues were kept in RNALater (Ambion, Carlsbad, CA, USA) prior to the RNA extraction. Total RNA was freshly isolated from the rat uteri using RNeasy Plus Mini Kit (Qiagen, Hilden, Germany). The purity and concentration of RNA was assessed by 260/280 UV absorption ratios (Gene Quant 1300, Cambidge, UK). Two steps Real Time PCR was used to evaluate gene expression with the application of TaqMan^®^ RNA-to-CT 1-Step Kit (Ambion, Carlsbad, CA, USA), which is highly sensitive [38]. Reverse transcription into cDNA was performed using High Capacity RNA-to-cDNA Kit (Applied Biosystems; Foster City, CA, USA). Controls include amplifications performed on the samples identically prepared with no reverse transcriptase (-RT) and amplifications performed with no added substrate (H_2_O control).

In real time PCR, the amplified region of the cDNA was probed with a fluorescence-labelled probe. The specificity of the primer and the probe ensures that the expression of the target DNA was specifically evaluated. Real time PCR does not require a time consuming post amplification gel electrophoresis due to its high sensitivity [37]. TaqMan probe has a sensitivity of 100% and specificity of 96.67% [38] and is capable of detecting as few as 50 copies of RNA/mL [39] and as low as 5–10 molecules [40]. The assay used (TaqMan^®^-catalogue number: 4351372 (Applied Biosystems, Foster City, CA, USA) amplifies 127 bp segment of SLC4A4 from the whole mRNA length of 3572 and 67 bp segment of SLC26A6 from the whole mRNA length of 2543 bp. Currently, there is no primer sequence that could differentiate between NBCe1-A and NBCe1-B, therefore specific mRNA expression analyses of these variants could not be performed. The target assay was validated *in silico* using whole rat genome sequences and *in vitro* using whole rat cDNA sequences to ensure target sequences were detected (Applied Biosystems, Foster City, CA, USA). Therefore, PCR product does not require additional sequencing for further validation. GAPDH was used as reference or house-keeping gene for the endometrial tissue as its expression was reported to be the most stable during the estrus cycle and in early pregnancy [41].

PCR program includes 2 min at 50 °C for Uracil *N*-glycosylase (UNG) activity, 20 s 95 °C activation of ampliTaq gold DNA polymerase, and 1 min denaturation at 95 °C, 20 s and annealing/extension at 60 °C for 1 min. Denature and annealing was performed for 40 cycles. All measurements were normalized using GenEx software (MultiD, Odingatan, Sweden) followed by Data Assist v3 software from Applied Biosystems (Applied Biosystems, Foster City, CA, USA). which was used to calculate the RNA folds changes. All experiments were carried out in three biological replicates. TaqMan^®^ (Applied Biosystems, Foster City, CA, USA) primers and probes for SLC26A6 and SLC4A4 and GAPDH were obtained from pre-designed assays (Applied Biosystems, Foster City, CA, USA). SLC26A6 and SLC4A4 assay numbers are Rn01445822 and Rn00670440-m1 respectively. The assay number for GAPDH is Rn99999916-s1. Data was analyzed according to the Comparative *C*t (2^−ΔΔ^*^C^*^t^) method. The major factor responsible for the accuracy of kinetic PCR method is determination of the *C*t value within the logarithmic phase of the amplification reaction [42], instead of endpoint determination by the conventional gel-based system. Calculation of *C*t value was made when amplification of the PCR products was first detected, after the beginning of the exponential phase of amplification. At this time, accumulation of inhibitory PCR products was unlikely to occur [43]. The relative quantity of the target in each sample was determined by comparing normalized target quantity in each sample to normalized target quantity of the reference gene.

### 3.4. SLC26A6, NBCe1-A and NBCe1-B Protein Expression Analysis by Western Blotting

Whole uterine tissues were snapped frozen in the liquid nitrogen and were then stored at −80 °C prior to protein extraction. Following total protein extraction with PRO-PREP solution (Intron, Seoul, Korea), an equal amount of protein from each tissue lysate were mixed with a loading dye, boiled for 5 min and separated using SDS-PAGE 12%. Protein was then transferred onto a PVDF membrane (BIORAD, Hercules, CA, USA) and blocked with 5% BSA for 90 min at room temperature. The membrane was exposed for 90 min to rabbit SLC26A6 and SLC4A4 (NBCe1-A and NBCe1-B) polyclonal primary antibodies (Santa Cruz: sc-26728 catalogue number: Abcam, Cambridge, UK: ab30322 and ab78326 respectively), at a dilution of 1:1000 in PBS containing 1% BSA and Tween-20. Membrane incubation with non-immune normal donkey and goat serum for SLC26A6 and SLC4A4 A and B respectively were used as a negative control and no band was observed in this experiment. The blots were then rinsed three times in PBS-T with each rinse lasted for five minutes. The membranes were then incubated with anti-rabbit, horseradish peroxidase conjugated secondary antibody (Santa Cruz, CA, USA) at a dilution of 1:2000, for 1 h. The membrane was then rinsed and subjected to Opti-4CN™ Substrate Kit (Bio-Rad, Hercules, CA, USA) to visualize the protein bands. Photos of the blots were captured by a gel documentation system and the density of each band was determined using Image J software NIH ImageJ (version 1.46j; National Institutes of Health, Bethesda, MD, USA). Ratio of each target band/β-actin was calculated and was considered as the expression level of the target proteins.

### 3.5. Detection of SLC26A6, NBCe1-A and NBCe1-B Distribution by Immuno-Histochemistry (IHC)

Uterine tissues were fixed overnight in 4% paraformaldehyde (PFD) before processing and dehydrated through increasing concentrations of ethanol, cleared in chloroform and were then blocked in the paraffin wax. For IHC, tissues were cut into 5 μm sections, deparaffinized in xylene, rehydrated in reducing concentration of ethanol. Tri sodium citrate (pH 6.0) was used for antigen retrieval, while 1% H_2_O_2_ in PBS was used to neutralize the endogenous peroxidase. Sections were then blocked in 5% BSA for the non-specific binding, prior to incubation with rabbit SLC26A6 and SLC4A4 (NBCe1-A and NBCe1-B) primary antibodies at a dilution of 1:100 in 5% BSA at room temperature for an hour. After four times rinsing with PBS, the sections were then incubated with biotinylated secondary antibody for 30 min at room temperature, and were then exposed to Avidin and biotinylated HRP complex (Santa Cruz, CA, USA) in PBS for another half an hour. The sites of antibody binding were visualized using DAB (Diaminobenzidine HCl) (Santa Cruz, CA, USA) which gave dark-brown stain. Sections were counterstained with hematoxylin for the nuclear staining.

In this experiment, non-immune normal donkey and goat serum for SLC26A6 and SLC4A4 A and B respectively were used as a negative control with no staining observed. Meanwhile, kidney section was used as a positive control where NBCe1-A immunostaining could be detected in the proximal tubules [44].

### 3.6. Quantifying Staining Intensity by NIS-Element AR Program

The slides were viewed using a Nikon Eclipse 80i microscope (SEO Enterprises Inc, Lakeland, FL, USA) with a Nikon DS Ri1 12 megapixel camera (Ningbo Jiangdong Deno International Trade Co., Ltd., Ningbo, China) attached. All images were captured under standard conditions of illumination. The voltage for illumination was chosen with the photographs taken at a fixed exposure time. Tiff images (1280 × 1024 pixels) were taken at objective lens magnification of 40× and 100×. Using NIS-Element AR program (SEO Enterprises Inc, Lakeland, FL, USA), the exposure time and sensitivity were set prior to image capturing. At the outset of the session, part of the slide with no tissue (blankfield) was viewed under the microscope and an auto white balance was carried out. Under the hue-saturation-intensity (HSI) mode, the area of interest on the image was selected and the total counts (spots with dark-brown stained) was obtained. The mean intensity of the counts (which can be restricted) was determined which represents the average amount of protein expressed in the tissue.

### 3.7. Statistical Analysis

Statistical differences were evaluated by analysis of variance (ANOVA) followed by Duncan’s new multiple-range test and Student’s *t*-test. A probability level of less than 0.05 (*p* < 0.05) was considered to be significantly different. *Post-hoc* statistical power analysis was performed for all the experiments conducted and all values obtained were >0.8 which were considered as adequate. Meanwhile, Shapiro-Wilk test was performed to test for data normality and all values obtained were >0.05 which indicated that these data were normally distributed. For the functional study, mRNA expression analyses, six amples were used while for Western blotting and immunohistochemistry, four samples were used per treatment group.

## 4. Conclusions

In conclusion, this study indicates that high dose of genistein (at 100 mg/kg/day) was able to induce changes in the uterine fluid pH, Na^+^ and HCO_3_^−^ concentration resemble the effect of estrogen. We have also shown the increased expression of SLC26A6 and NBCe1-B at the apical membrane of the endometrial epithelia under high dose genistein effect could explain the observed changes in these uterine fluid parameters, thus may contribute to the reported infertility associated with high genistein consumption.

## References

[1] Al-Nakkash L., Clarke L.L., Rottinghaus G.E., Chen Y.J., Cooper K., Rubin L.J. (2006). Dietary genistein stimulates anion secretion across female murine intestine. J. Nutr.

[2] Illek B., Yankaskas J.R., Machen T.E. (1997). cAMP and genistein stimulate HCO_3_^−^ conductance through CFTR in human airway epithelia. Am. J. Physiol.

[3] Tuo B., Wen G., Seidler U. (2009). Differential activation of the HCO_3_^−^ conductance through the cystic fibrosis transmembrane conductance regulator anion channel by genistein and forskolin in murine duodenum. Br. J. Pharmacol.

[4] Chao P.-C., Hamilton K.L. (2009). Genistein stimulates electrogenic Cl^−^ secretion via phosphodiesterase modulation in the mouse jejunum. Am. J. Physiol. Cell Physiol.

[5] Leung G.P.H., Wong P.Y.D. (2000). Activation of cystic fibrosis transmembrane conductance regulator in rat epididymal epithelium by genistein. Biol. Reprod.

[6] Abdulnour-Nakhoul S., Nakhoul H.N., Kalliny M.I., Gyftopoulos A., Rabon E., Doetjes R., Brown K., Nakhoul N.L. (2011). Ion transport mechanisms linked to bicarbonate secretion in the esophageal submucosal glands. Am. J. Physiol. Regul. Integr. Comp. Physiol.

[7] He Q., Chen H., Wong C.H., Tsang L.L., Chan H.C. (2010). Regulatory mechanism underlying cyclic changes in mouse uterine bicarbonate secretion: Role of estrogen. Reproduction.

[8] Gholami K., Muniandy S., Salleh N. (2013). *In vivo* functional study on the involvement of CFTR, SLC26A6, NHE-1 and CA isoenzymes II and XII in uterine fluid pH, volume and electrolyte regulation in rats under different sex-steroid influence. Int. J. Med. Sci.

[9] Vincourt J.B., Jullien D., Kossida S., Amalric F., Girard J.P. (2002). Molecular cloning of SLC26A7, a novel member of the SLC26 sulfate/anion transporter family, from high endothelial venules and kidney. Genomics.

[10] Lohi H., Kujala M., Kerkelä E., Saarialho-Kere U., Kestilä M., Kere J. (2000). Mapping of five new putative anion transporter genes in human and characterization of SLC26A6, a candidate gene for pancreatic anion exchanger. Genomics.

[11] Chernova M.N., Jiang L., Friedman D.J., Darman R.B., Lohi H., Kere J., Vandorpe D.H., Alper S.L. (2005). Functional comparison of mouse slc26a6 anion exchanger with human SLC26A6 polypeptide variants: Differences in anion selectivity, regulation, and electrogenicity. J. Biol. Chem.

[12] Xie Q., Welch R., Mercado A., Romero M.F., Mount D.B. (2002). Molecular characterization of the murine Slc26a6 anion exchanger: Functional comparison with Slc26a1. Am. J. Physiol. Ren. Physiol.

[13] Petrovic S., Ma L., Wang Z., Soleimani M. (2003). Identification of an apical exchanger in rat kidney proximal tubule. Am. J. Physiol. Cell Physiol.

[14] Säfsten B. (1993). Duodenal bicarbonate secretion and mucosal protection. Neurohumoral influence and transport mechanisms. Acta Physiol. Scand. Suppl.

[15] Ishiguro H., Yamamoto A., Nakakuki M., Yi L., Ishiguro M., Yamaguchi M., Kondo S., Mochimaru Y. (2012). Physiology and pathophysiology of bicarbonate secretion by pancreatic duct epithelium. Nagoya J. Med. Sci.

[16] Abuladze N., Lee I., Newman D., Hwang J., Boorer K., Pushkin A., Kurtz I. (1998). Molecular cloning, chromosomal localization, tissue distribution, and functional expression of the human pancreatic sodium bicarbonate cotransporter. J. Biol. Chem.

[17] Romero M.F., Chen A.P., Parker M.D., Boron W.F. (2013). The SLC4 family of bicarbonate (HCO_3_^−^) transporters. Mol. Aspects Med.

[18] Shahidullah M., To C.H., Pelis R.M., Delamere N.A. (2009). Studies on bicarbonate transporters and carbonic anhydrase in porcine nonpigmented ciliary epithelium. Investig. Ophthalmol. Vis. Sci.

[19] Oehlke O., Speer J.M., Roussa E. (2013). Variants of the electrogenic sodium bicarbonate cotransporter 1 (NBCe1) in mouse hippocampal neurons are regulated by extracellular pH changes: Evidence for a Rab8a-dependent mechanism. Int. J. Biochem. Cell Biol.

[20] Kurtz I., Zhu Q. (2013). Structure, function, and regulation of the SLC4 NBCe1 transporter and its role in causing proximal renal tubular acidosis. Curr. Opin. Nephrol. Hypertens.

[21] Yang D., Shcheynikov N., Zeng W., Ohana E., So I., Ando H., Mizutani A., Mikoshiba K., Muallem S. (2009). IRBIT coordinates epithelial fluid and HCO_3_^−^ secretion by stimulating the transporters pNBC1 and CFTR in the murine pancreatic duct. J. Clin. Investig.

[22] Bucking C., Wood C. (2012). Digestion of a single meal affects gene expression of ion and ammonia transporters and glutamine synthetase activity in the gastrointestinal tract of freshwater rainbow trout. J. Comp. Physiol. B.

[23] Lee S.-K., Boron W.F., Parker M.D. (2012). Relief of autoinhibition of the electrogenic Na-HCO_3_ cotransporter NBCe1-B: Role of IRBIT *vs.* amino-terminal truncation. Am. J. Physiol. Cell Physiol.

[24] Suzuki M., Seki G., Yamada H., Horita S., Fujita T. (2012). Functional roles of electrogenic sodium bicarbonate cotransporter NBCe1 in ocular tissues. Open Ophthalmol. J.

[25] Nguyen T.T., Bonanno J.A. (2011). Bicarbonate, NBCe1, NHE, and carbonic anhydrase activity enhance lactate-H^+^ transport in bovine corneal endothelium. Investig. Ophthalmol. Vis. Sci.

[26] Zin S.R.M., Omar S.Z., Khan N.L., Musameh N.I., Das S., Kassim N.M. (2013). Effects of the phytoestrogen genistein on the development of the reproductive system of Sprague Dawley rats. Clinics.

[27] Li H.F., Duan Y., Wang L.D., Tian Z.F., Qiu X.Q., Zhang Y.F., Zhang H., Yang L.N. (2013). Effects of estrogen and phytoestrogens on endometrial leakage in ovariectomized rats and the related mechanisms. Sheng Li Xue Bao.

[28] Tuo B., Wen G., Song P., Xu J., Liu X., Seidler U., Dong H. (2011). Genistein stimulates duodenal HCO_3_^−^ secretion through PI3K pathway in mice. Eur. J. Pharmacol.

[29] Singh A.K., Liu Y., Riederer B., Engelhardt R., Thakur B.K., Soleimani M., Seidler U.E. (2013). Molecular transport machinery involved in orchestrating luminal acid-induced duodenal bicarbonate secretion *in vivo*. J. Physiol..

[30] Stewart A.K., Yamamoto A., Nakakuki M., Kondo T., Alper S.L., Ishiguro H. (2009). Functional coupling of apical Cl^−^/HCO_3_^−^ exchange with CFTR in stimulated HCO_3_^−^ secretion by guinea pig interlobular pancreatic duct. Am. J. Physiol. Gastrointest. Liver Physiol.

[31] Little D.L. (1996). Reducing the effects of clover disease by strategic grazing of pastures. Aust. Vet. J.

[32] Setchell K.D., Gosselin S.J., Welsh M.B., Johnston J.O., Balistreri W.F., Kramer L.W., Dresser B.L., Tarr M.J. (1987). Dietary estrogens—A probable cause of infertility and liver disease in captive cheetahs. Gastroenterology.

[33] Salleh N., Helmy M.M., Fadila K.N., Yeong S.O. (2013). Isoflavone genistein induces fluid secretion and morphological changes in the uteri of post-pubertal rats. Int. J. Med. Sci.

[34] Chan H.C., Chen H., Ruan Y., Sun T. (2012). Physiology and pathophysiology of the epithelial barrier of the female reproductive tract: Role of ion channels. Adv. Exp. Med. Biol.

[35] Vuento M.H., Pirhonen J.P., Mäkinen J.I., Tyrkkö J.E., Laippala P.J., Grönroos M., Salmi T.A. (1996). Endometrial fluid accumulation in asymptomatic postmenopausal women. Ultrasound Obstet. Gynecol.

[36] Salleh N., Baines D.L., Naftalin R.J., Milligan S.R. (2005). The hormonal control of uterine luminal fluid secretion and absorption. J. Membr. Biol.

[37] Hofmann-Lehmann R., Swenerton R.K., Liska V., Leutenegger C.M., Lutz H., McClure H.M., Ruprecht R.M. (2000). Sensitive and robust one-tube real-time reverse transcriptase-polymerase chain reaction to quantify SIV RNA load: Comparison of one- *vs.* two-enzyme systems. AIDS Res. Hum. Retrovir.

[38] Tsai Y.-L., Wang H.-T.T., Chang H.-F.G., Tsai C.-F., Lin C.-K., Teng P.-H., Su C., Jeng C.-C., Lee P.-Y. (2012). Development of TaqMan probe-based insulated isothermal PCR (iiPCR) for sensitive and specific on-site pathogen detection. PLoS One.

[39] Leutenegger C., Higgins J., Matthews T.B., Tarantal A.F., Luciw P.A., Pedersen N.C., North T.W. (2001). Real-time TaqMan PCR as a specific and more sensitive alternative to the branched-chain DNA assay for quantitation of simian immunodeficiency virus RNA. AIDS Res. Hum. Retrovir.

[40] Hofmann-Lehmann R., Williams A.L., Swenerton R.K., Li P.L., Rasmussen R.A., Chenine A.L., McClure H.M., Ruprecht R.M. (2002). Quantitation of simian cytokine and beta-chemokine mRNAs, using real-time reverse transcriptase-polymerase chain reaction: Variations in expression during chronic primate lentivirus infection. AIDS Res. Hum. Retrovir.

[41] Lin P., Lan X., Chen F., Yang Y., Jin Y., Wang A. (2013). Reference gene selection for real-time quantitative PCR analysis of the mouse uterus in the peri-implantation period. PLoS One.

[42] Pfaffl M.W. (2001). A new mathematical model for relative quantification in real-time RT-PCR. Nucleic Acids Res.

[43] Schmittgen T., Livak K.J. (2008). Analyzing real-time PCR data by the comparative *C*_t_ method. Nat. Protoc.

[44] Zhu Q., Liu W., Kao L., Azimov R., Newman D., Abuladze N., Kurtz I. (2013). Topology of NBCe1 protein transmembrane segment 1 and structural effect of proximal renal tubular acidosis (pRTA) S427L mutation. J. Biol. Chem.

